# Supplementary material for: MetaRibo-Seq measures translation in microbiomes
Source: Nat Commun. 2020 Jun 29;11:3268. doi: 10.1038/s41467-020-17081-z (PMC7324362; doi:10.1038/s41467-020-17081-z)
Supplement: Supplementary file 10 — Supplementary Data 7 [file 41467_2020_17081_MOESM10_ESM.zip › File2/Confidence_High_Taxonomy/308104_out.krona.html]

Javascript must be enabled to view this page.

members
magnitude
magnitudeUnassigned
count
unassigned
taxon
rank

308104\_out

20

20
2
superkingdom

1239
20
phylum

186801
20
class

20
5

SRS013098\_contig\_number\_contig-100\_8631.227494SRS077730\_contig\_number\_14717SRS1041095\_contig\_number\_contig-100\_1385.1386SRS147022\_contig\_number\_13720SRS147139\_contig\_number\_6294
186802
order

1392389
2
genus

species

SRS015190\_contig\_number\_contig-100\_16181.16181SRS1054691\_contig\_number\_9582
2
1297617

541000
11
family

10
946234
genus

10
292800
species

SRS046712\_contig\_number\_1394SRS063127\_contig\_number\_1009SRS077502\_contig\_number\_11843SRS143991\_contig\_number\_5106SRS146764\_contig\_number\_25016SRS146888\_contig\_number\_3664SRS148091\_contig\_number\_contig-100\_8502.8503SRS148511\_contig\_number\_contig-100\_4610.88318SRS893253\_contig\_number\_contig-100\_2100.24714SRS893256\_contig\_number\_contig-100\_2336.26145

genus
1
216851

1971605
1

SRS049402\_contig\_number\_9324
species

31979
2
family

genus
2
1485

species

SRS057478\_contig\_number\_contig-100\_13791.13792SRS098655\_contig\_number\_3654
2
649724
